# Fair access to medicine? Retrospective analysis of UK medical schools application data 2009-2012 using three measures of socioeconomic status

**DOI:** 10.1186/s12909-016-0536-1

**Published:** 2016-01-13

**Authors:** Kathryn Steven, Jon Dowell, Cathy Jackson, Bruce Guthrie

**Affiliations:** School of Medicine, University of Dundee, Dundee, Scotland; School of Medicine, University of Central Lancashire, Preston, England; Population Health Sciences Division, School of Medicine, University of Dundee, Dundee, Scotland

**Keywords:** Selection, Socio-economic status, Education, Widening participation, Deprivation

## Abstract

**Background:**

Medical students have historically largely come from more affluent parts of society, leading many countries to seek to broaden access to medical careers on the grounds of social justice and the perceived benefits of greater workforce diversity. The aim of this study was to examine variation in socioeconomic status (SES) of applicants to study medicine and applicants with an accepted offer from a medical school, comparing the four UK countries and individual medical schools.

**Methods:**

Retrospective analysis of application data for 22 UK medical schools 2009/10-2011/12. Data were analysed for all 32,964 UK-domiciled applicants aged <20 years to 22 non-graduate medical schools requiring applicants to sit the United Kingdom Clinical Aptitude Test (UKCAT). Rates of applicants and accepted offers were compared using three measures of SES: (1) Postcode-assigned Index of Multiple Deprivation score (IMD); (2) School type; (3) Parental occupation measured by the National Statistics Socio Economic Classification (NS-SEC).

**Results:**

There is a marked social gradient of applicants and applicants with accepted offers with, depending on UK country of residence, 19.7–34.5 % of applicants living in the most affluent tenth of postcodes vs 1.8–5.7 % in the least affluent tenth. However, the majority of applicants in all postcodes had parents in the highest SES occupational group (NS-SEC1). Applicants resident in the most deprived postcodes, with parents from lower SES occupational groups (NS-SEC4/5) and attending non-selective state schools were less likely to obtain an accepted offer of a place at medical school further steepening the observed social gradient. Medical schools varied significantly in the percentage of individuals from NS-SEC 4/5 applying (2.3 %–8.4 %) and gaining an accepted offer (1.2 %–7.7 %).

**Conclusion:**

Regardless of the measure, those from less affluent backgrounds are less likely to apply and less likely to gain an accepted offer to study medicine. Postcode-based measures such as IMD may be misleading, but individual measures like NS-SEC can be gamed by applicants. The previously unreported variation between UK countries and between medical schools warrants further investigation as it implies solutions are available but inconsistently applied.

## Background

Over 100 years ago Flexner wrote ‘We have no right… to set up standards which will close the profession to “poor boys” [1]’. However, medicine in the UK remains dominated by those from more affluent backgrounds, and policy and professional stakeholders internationally identify widening participation as a significant issue facing the medical profession [2–7].

Widening participation has been defined as ‘ensuring that students from disadvantaged backgrounds can access higher education, get the support they need to succeed in their studies, and progress to further study and/or employment suited to their qualifications and potential’ [8]. Recruitment from disadvantaged groups is not a problem confined to UK medical schools. In the USA, there is a persistent decline in applicants and matriculants from the Black and African American male population along with a recognised need for continued work in recruitment from under-represented ethnic and racial groups [9]. In Australia, recruitment to undergraduate medical degrees from indigenous populations is increasing but remains low [10]. In the UK, female and ethnic minority groups are now well represented in medicine, but those from a lower socio-economic status remain under-represented (SES). In 2012, the Social Mobility and Child Poverty Commission reported that within the UK, the proportion of medical students from private schools was not changing, that the responses from professional bodies regarding widening participation (WP) were poor and ultimately concluded that “medicine has made far too little progress” [11].

The most common argument for widening participation is on the grounds of social justice and the wider desirability of improving social mobility. Other benefits may include the development of a more diverse workforce providing better care for the whole population. The evidence for this is mostly from the USA, where for example, physicians from ethnic minority groups are more likely to work in underserved communities and with patients of the same ethnicity [12, 13]. Studying in a more diverse medical school also has positive effects on students’ attitudes towards diversity related issues and may increase preparedness to care for minority groups within society [14, 15]. In Scotland, general practitioners from less affluent backgrounds have been shown to be more likely to work in practices serving the most deprived communities [16]. Research from the USA also suggests that students from lower income groups may be more likely to pursue family medicine, which is a priority in many countries, including the UK where the government has proposed that 50 % of foundation year trainees should enter GP speciality training [17] in order to allow health services to effectively manage the growing number of older people with multimorbidity [18].

Despite the policy focus, it remains unclear how best to measure whether the social mix of medical students according to SES is changing as the result of widening participation activity. The most common measures used in the UK rely on postcode assigned SES such as the Index of Multiple Deprivation (IMD, a small area measure of SES routinely used in UK health services research as a proxy for individual SES) or on school type (selective or not, state vs. independent/fee paying) which are relatively easy to measure. These are area or institutional variables and so subject to ecological fallacy when applied to individuals. They are also not comparable across the UK, since IMD measurement is country specific [19, 20], and the four UK countries (England, Northern Ireland, Scotland, Wales) have different schooling systems which vary both in the proportion of children attending independent schools and the degree of selection in the state system. Individual measures, such as parental National Statistics Socio-Economic Classification of occupation (NS-SEC) and assessment of household income avoid the problems of area-based measures, but would be complex to measure on a large scale and are more open to gaming by applicants if explicitly used in selection.

The aim of this study is to examine how UK secondary school leaver applicants to medical school and those with an ‘accepted offer’ of a place vary by SES using two measures in widespread use (postcode-assigned IMD score and school type) and one individual measure (parental NS-SEC).

## Methods

### Dataset

Applicants to medical school in the UK apply through the Universities and Colleges Admissions System (UCAS) and most UK medical schools also require applicants to take one of two specific aptitude tests. Over the period examined, 25 (78 %) medical schools required applicants to sit the United Kingdom Clinical Aptitude test (UKCAT). Linked UCAS and UKCAT data were used to examine the SES of applicants to, and those with an accepted offer from 22 UKCAT Consortium Schools.

### Population

Data from three admission cycles were examined (2009–2010 to 2011–2012). An admission cycle crosses calendar years since an applicant sits the UKCAT and makes their UCAS application in one year for medical school entry in the next calendar year at the earliest. Widening participation in the UK is primarily framed in terms of the family and social-economic background of applicants. From this perspective, it is relatively difficult to account for graduate entrants, whose current individual SES based on any measure may or may not reflect their family background. This analysis is therefore of UK domiciled applicants aged 19 or under at the time of application, who applied to at least one of 22 UKCAT Consortium medical schools (three graduate-entry schools were excluded). For all included applicants, data are available on whether the individual received an offer from each of the UKCAT medical schools they applied to, and whether they firmly accepted that offer.

### Measures of SES

Three measures of SES were defined. First, the Index of Multiple Deprivation (IMD) was calculated for the postcode of residence recorded in the UCAS application. IMD is a weighted score of a number of indicators of SES and ranks small areas in order of deprivation. IMD is not an individual measure of SES, since an affluent individual may live in a deprived area and vice versa. Each UK country has its own IMD measure, and although they are similar, there is variation in the indicators used, the weighting assigned to each indicator, the frequency of updating, and the size of the geography being measured (from ~750 residents in Scotland to ~2000 in Northern Ireland) [20]. IMD scores are therefore not strictly comparable across countries and the Office of National Statistics recommends not using IMD as a UK wide measure [20], although IMD is often treated as such. For each country, small areas defined by postcode were ranked in ascending order of affluence, and categorised into centiles (equally sized hundredths) and deciles (equally sized tenths, where centile/decile 1 represents the least affluent group).

Second, data from the UCAS application were used to define the school type an applicant attended. Like IMD, school type is a proxy rather than an individual measure of SES, but because it is routinely collected, it is also commonly used to examine participation. The state schooling systems in the four UK countries differ. In England, following the Direct Grant Grammar Schools Regulations of 1975 many grammar schools were closed, but some English counties retain significant numbers of state-funded selective grammar schools, which have a higher percentage of students from more affluent backgrounds [21]. Selective schools are those which require potential students to sit an entrance examination with selection and admission to the school based on the individual’s performance. Scotland and Wales no longer have a selective grammar system. Northern Ireland has attempted to move away from selective secondary schooling and no longer uses the ‘transfer test’ to determine secondary school entry at age 11 years, although most secondary schools continue to set their own entrance exam. Given the differences in interpretation of state school type between countries, and the public focus on independent vs. state schools when considering widening participation, we defined school type as independent (fee-paying) school, grammar school (selective entrance exam, state funded), or a non-selective state funded school (comprehensive, sixth form college, or further education college). In the UK as a whole, only 6.5 % of children are educated in the independent sector [22].

Third, for each candidate we calculated parental National Statistics Socio-Economic Classification (NS-SEC) based on applicant responses to the self-report questions used by the Office of National Statistics (ONS) [23] undertaken during the UKCAT registration process. Applicants were allocated to one of five NS-SEC groups based on the highest NS-SEC of either parent. NS-SEC differs from the previous Registrar General Classification of occupation. Firstly, it is not strictly linear. NS-SEC 1 (higher managerial, administrative and professional occupations) and NS-SEC 2 (intermediate occupations) can be considered higher SES and NS-SEC 4 (lower supervisory and technical occupations) and NS-SEC 5 (semi-routine and routine occupations) lower SES, but NS-SEC 3 (small employers and own account workers) is a distinct occupational group which is not ordered in the same way. Secondly when considering the most affluent group in both classification systems, NS-SEC 1 is a broader, numerically larger group than the previous social class 1, including both traditional professional occupations like medicine and law and modern professional occupations like nursing and teaching, as well as senior management. The proportion of the UK population in the five categories in 2011 was NS-SEC 1 41.4 %, NS-SEC 2 12.7 %, NS-SEC 3 9.4 %, NS-SEC 4 6.9 % and NS-SEC 5 25.2 % [24]. Unlike IMD and school type, NS-SEC is an individual measure of SES, but completion of the NS-SEC questions during registration is voluntary so this information is not available for all applicants with approximately 10 % of data missing for school-leaver applicants in the period examined.

### Data analysis

For each country and for the UK as a whole, we calculated the percentage of applicants from each decile of IMD score (equal tenths of postcodes ranked in ascending order of affluence), each school type and each NS-SEC category. For IMD and NS-SEC, we then estimated an ‘application ratio’ which is the ratio of the proportion of applicants in each subcategory to the expected proportion of the population in that subcategory (akin to the standardised admission ratio proposed by Seyan et al) [25]. This process was repeated for the percentage of applicants who received an accepted offer, with a ‘selection ratio’ estimated which is the ratio of the proportion of applicants with an accepted offer in each subcategory to the proportion of all applicants in that subcategory. To further examine the distribution of applicant by SES, we calculated the percentage of applicants and the percentages of applicants with an accepted offer for 100 equally sized groups of postcodes ranked in ascending order of affluence by IMD, and examined how school type and NS-SEC were distributed across the range of IMD. Finally, we defined a single binary measure of participation by applicants from less affluent families as an applicant having parents in NS-SEC 4 or 5 (lower supervisory and technical, and semi-routine and routine occupations), and examined how the percentage of applicants and accepted offers made to this group varied between the 22 medical schools. Data were analysed in a web-based safe haven managed by the Health Informatics Centre, University of Dundee, under data governance rules established by the UKCAT Consortium. The study was reviewed and approved by the University of St Andrews Teaching and Research Ethics Committee. Analysis was carried out in IBM SPSS v11.

## Results

There were 32,964 UK domiciled applicants aged 19 or under to one or more of the 22 UKCAT Consortium medical schools for the three admission cycles 2009–10, 2010–11 and 2011–12. 32,065 (97.3 %) had a postcode from which IMD could be calculated and were included in the analysis. 28,902 (90.1 % of those included) had a valid parental NS-SEC and 31,028 (96.8 % of those included) had a valid school type. Rates of missing data varied across countries, from 4.9 to 10.8 % for NS-SEC (highest for England) and 2.9 to 5.0 % for school type (highest for Scotland). 10,437 (32.5 %) applicants accepted an offer of a place from any UKCAT medical school.

### IMD centile

**Fig. 1:**
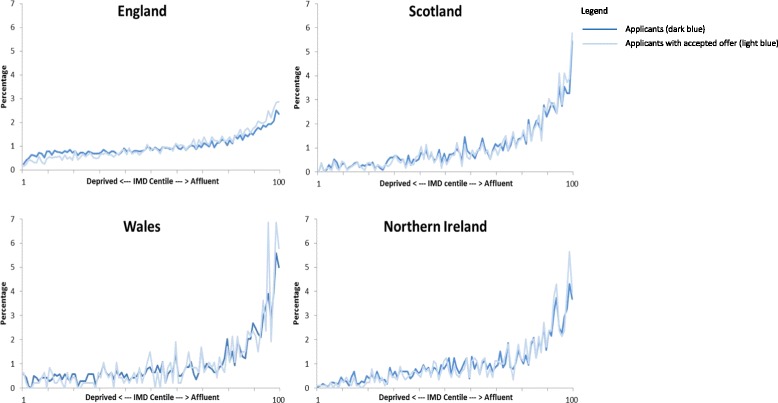
Percentage of applicants and applicants with an accepted offer by centile of IMD in 2009–2010 to 2011–2012 admission cycles

Figure 1 shows the percentage of applicants and applicants with accepted offers resident in each centile of postcodes ranked in ascending order of affluence (1 = deprived, 100 = affluent). In all four UK countries, there is a marked and consistent social gradient with fewer applicants and applicants with accepted offers resident in less affluent postcodes. Across the four UK countries, 19.7 to 34.5 % of applicants lived in the most affluent decile of postcodes compared to 1.8 to 5.7 % of applicants living in the least affluent decile (Table 1). The application ratio across the whole of the UK increased progressively by IMD decile from 0.51 for the most deprived decile (half the applicants expected) to 2.19 for the most affluent (twice the applicants expected). Overall England did appear to have the least inequitable distribution of applicants (although IMD rankings are not strictly comparable between countries).

**Table 1:** Percentage of applicants to medical school by UK country according to four measures of socioeconomic status (SES)

	England (*n* = 26442)	Scotland (*n* = 2479)	Wales (*n* = 1380)	N. Ireland (*n* = 1764)	UK (*n* = 32065)	UK application ratio (applicants: estimated population)
IMD decile (postcode assigned SES)						
1 deprived	5.7	2.3	3.6	1.8	5.1	0.51
2	7.6	2.7	4.6	3.2	6.9	0.69
3	7.1	3.3	3.9	4.6	6.6	0.66
4	7.7	5.0	6.1	6.2	7.3	0.73
5	8.4	6.8	5.7	7.7	8.1	0.81
6	9.2	8.0	7.6	9.0	9.0	0.90
7	9.5	8.9	7.8	9.2	9.4	0.94
8	11.3	11.9	9.9	11.7	11.3	1.13
9	13.8	18.9	16.4	16.2	14.5	1.45
10 affluent	19.7	32.3	34.5	30.4	21.9	2.19
School type^a^						
Independent	25.9	29.6	14.9	0.2	24.3	3.74
State	71.1	65.4	82.1	96.7	72.5	0.78
*Grammar*	*19.5*	*0.1*	*0.1*	*91.0*	*21.1*	-
*Comp/FEC/SFC*	*51.6*	*65.3*	*82.0*	*5.7*	*51.4*	-
Missing/Unknown/Other	3.1	5.0	2.9	3.1	3.2	-
Parental NS-SEC						
1 Higher managerial/admin & professional	73.9	83.7	80.7	79.6	75.3	2.0
2 Intermediate	4.6	5.0	5.2	5.6	4.7	0.4
3 Small employers, own account workers	5.6	3.8	3.9	5.3	5.4	0.6
4 Lower supervisory and technical	1.9	1.5	1.7	2.7	1.9	0.3
5 Semi-routine and routine	3.2	1.0	1.8	1.5	2.9	0.1
Missing	10.8	4.9	6.7	5.3	9.9	-

^a^Independent schools are fee-paying and mainly select by academic ability. State schools are government funded and free to use. Grammar schools select by academic ability. Comp/FEC/Other refers to comprehensive schools (non-selective), further education colleges (which provide non-selective education to some 16–18 year olds), other refers to a range of other school types including sixth-form colleges (which only provide education to 16–18 year olds)

Between 22.9 and 37.7 % of applicants with accepted offers lived in the most affluent decile, compared to 1.2 to 3.5 % from the least affluent decile (Table 2). Again, there was a consistent social gradient with accepted offer: applicant ratio ranging from 0.6 for the most deprived decile to 1.19 for the most affluent.

**Table 2:** Percentage of applicants with an accepted offer for medical school by UK country according to four measures of socioeconomic status (SES)

	England (*n* = 7772)^1^	Scotland (*n* = 1315)	Wales (*n* = 467)^1^	N. Ireland (*n* = 883)	UK (*n* = 10437)	UK selection ratio (accepted offers: applicants)
IMD decile (postcode assigned SES)						
1 deprived	3.5	1.9	2.6	1.2	3.1	0.60
2	5.2	2.4	3.9	2.0	4.5	0.66
3	5.9	2.8	2.1	4.0	5.2	0.79
4	6.9	4.6	5.4	5.5	6.4	0.88
5	7.9	6.5	6.2	7.6	7.6	0.94
6	9.1	6.7	6.9	7.5	8.5	0.95
7	10.6	8.7	8.4	9.3	10.1	1.08
8	12.5	12.1	9.4	11.9	12.3	1.09
9	15.4	18.9	17.6	17.4	16.1	1.11
10 affluent	22.9	35.5	37.7	33.5	26.1	1.19
School type^a^						
Independent	29.0	34.8	13.9	0.1	26.6	1.09
State	68.2	61.0	82.2	96.3	70.3	0.97
*Grammar*	*22.2*	*0.1*	*0.2*	*92.1*	*24.3*	1.15
*Comp/FEC/SFC*	*46.0*	*60.9*	*82.0*	*4.2*	*46.0*	0.89
Missing/Unknown/Other	2.9	4.2	3.9	3.6	3.1	0.97
Parental NS-SEC						
1 Higher managerial/admin & professional	78.3	86.2	83.9	81.4	79.8	1.06
2 Intermediate	4.3	4.6	4.1	5.5	4.4	0.94
3 Small employers, own account workers	4.7	3.1	3.4	5.0	4.5	0.83
4 Lower supervisory and technical	1.6	1.4	0.6	1.7	1.5	0.79
5 Semi-routine and routine	2.0	0.6	1.9	0.9	1.7	0.59
Missing	9.2	4.0	6.0	5.4	8.1	0.82

^a^Independent schools are fee-paying and mainly select by academic ability. State schools are government funded and free to use. Grammar schools select by academic ability. Comp/FEC/Other refers to comprehensive schools (non-selective), further education colleges (which provide non-selective education to some 16–18 year olds), other refers to a range of other school types including sixth-form colleges (which only provide education to 16–18 year olds

### School type

**Fig. 2:**
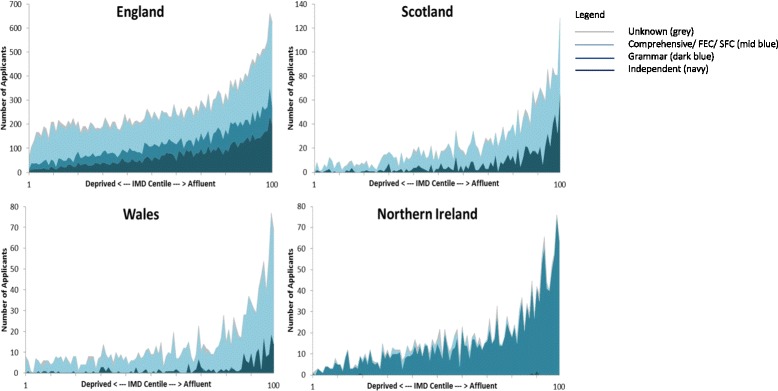
School type by centile of IMD for all applicants in 2009–2010 to 2011–2012 admission cycles

The application ratio for the UK for independent school applicants is 3.74 (three times the number of applicants expected) and for state schools 0.78 (about three quarters of the applicants expected). English (25.9 %) and Scottish (29.6 %) applicants were more likely to attend independent schools than Welsh (14.9 %) or Northern Irish (0.2 %) (Table 1). Applicants from independent and grammar schools were slightly more likely to receive an accepted offer than average, with accepted offer: applicant ratios of 1.09 and 1.15 respectively (Table 2). Figure 2 shows the distribution of school type among applicants from each IMD centile, the differences demonstrate why school type is an inconsistent measure to use within the UK. Applicants attending independent schools were more likely to live in more affluent postcodes, but were distributed across the entire distribution of IMD.

### NS-SEC

**Fig. 3:**
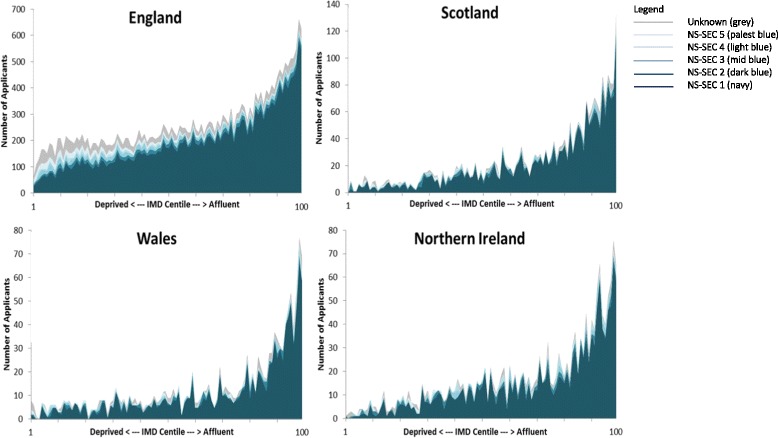
Parental NS-SEC by centile of IMD for all applicants in 2009-2010 to 2011-2012 admission cycles

Approximately three-quarters of applicants in all four UK countries had at least one parent in NS-SEC 1, ranging from 73.9 % in England to 83.7 % in Scotland (Table 1). Only 1.9 % of applicants across the UK had parents in NS-SEC 4 and only 2.9 % from NS-SEC 5 despite the fact that the NS-SEC 5 group accounts for 25.2 % of the population (Table 1). The application ratio for NS-SEC 1 was 2.0 (twice the applicants expected) and for NS-SEC 5 the ratio was 0.1 (one tenth of the applicants expected). 79.8 % of applicants with accepted offers had parents in NS-SEC 1 group, and this group has an accepted offer: applicant ratio of 1.06. The ratio in NS-SEC 4 and 5 groups were 0.79 and 0.59 respectively (Table 2). Figure 3 shows the distribution of applicants to medical school within each IMD centile by NS-SEC. Strikingly, those with parents in NS-SEC 1 are the predominant applicant group across the spectrum of IMD centiles from deprived to affluent.

### Medical schools

**Fig. 4:**
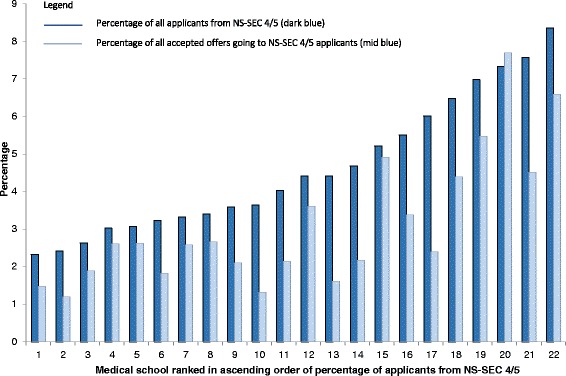
The percentage of NS-SEC 4/5 applicants and applicants with accepted offers out of all applicants / all accepted offers for 22 UKCAT Consortium Medical Schools

There was a near fourfold variation in the percentage of applicants to the 22 medical schools who had parents from NS-SEC 4 or 5, from 2.3 % (medical school 1) to 8.4 % (medical school 22), and six-fold variation in the percentage of applicants with an accepted offer with parents from NS-SEC 4 or 5, from 1.2 % (medical school 2) to 7.7 % (medical school 20) (Fig. 4). The percentage of applicants with accepted offers who had parents in NS-SEC 4 or 5 was highly correlated with the percentage of applicants from these groups (Pearson correlation coefficient 0.842, *p* < 0.001), but schools did vary significantly in the proportion of the NS-SEC 4 or 5 applicant group to their school who were given an offer and then accepted that offer with a range of 2.5 % to 26.1 % (median 8.0 %, interquartile range 6.5 %).

## Discussion

Postcode-assigned IMD is the most commonly used measure of SES in this context in the UK, but may be very misleading, since the majority of applicants had at least one parent in NS-SEC 1 across the entire distribution of IMD [26]. It is also notable that the observed social gradients in terms of the application ratio are much larger for the individual NS-SEC measure than postcode-derived IMD and school type, indicating that the latter two are likely to overestimate how wide participation is. However, irrespective of the measure used, it is clear that school-leaver applicants to medical courses are predominately drawn from the more affluent, and this largely drives the distribution of applicants with accepted offers. There are marked differences between medical schools in terms of the mix of applicants they attract, with almost a fourfold variation in the proportion of applicants from NS-SEC 4/5. Selection processes do therefore matter, but there will not be large-scale widening participation in medicine in the UK unless applicants come from a broader range of the population. There are also differences in applicants and those with accepted offers between the UK countries, with England appearing to have a more equitable distribution than the other three countries (although differences in IMD and school systems between countries may at least partly account for this).

This analysis shows that the dominance of medicine by the more affluent [8, 11, 27] is persistent despite increasingly intense activity over the last decade intended to widen participation [2]. Direct comparisons over time are difficult because SES measures have changed, but new measures like IMD and NS-SEC have the same problems as older measures. Do and Parry’s examination of English medical school applicants between 1996 and 2003 using older measures of SES (Townsend Score and Registrar General Classification) demonstrated that 60 % of applicants were from the professional/managerial class, and that while there was a positive association between parental occupation and Townsend score this was far from perfect [28].

A strength of the study is the use of linked UCAS and UKCAT data, ensuring that multiple measures of SES were available for the majority of medical schools and a large pool of applicants. Limitations include that the analysis presented does not account for ethnicity or gender. The interaction between ethnicity, gender and SES is of interest but was not the focus of this study, reflecting that widening participation in the UK is largely framed in terms of SES. The findings are consistent with low participation being largely driven by low rates of application from the least affluent, although this will partly reflect variation in academic achievement by SES which was not examined [29]. The analysis also shows that the mix of applicants and selection outcomes vary between medical schools implying that widening participation is possible, and previous analyses have shown that medical schools that more actively use UKCAT test scores in selection admit more applicants from lower SES backgrounds [30]. Eight UK medical schools were not in the UKCAT Consortium during the period of analysis, although in practice a large proportion of applicants will apply to at least one UKCAT Consortium school and will therefore be included. Finally, the analysis is only of UK domiciled ‘secondary school leaver’ applicants. However, measuring SES in graduate entrants is fraught with difficulty, because from a widening participation perspective it is childhood or family SES rather than current SES which is of interest, but existing data collection systems are based on current, individual postcode or income. Additionally, Mathers et al in their analysis of successful applicants to medical school between 2002 and 2006 reported the socio-economic profile of medical students did not seem to have been affected by graduate entry programs [31].

There are two key implications of this research. First, modifying selection processes is unlikely to have major impact on widening participation because so few people from less affluent backgrounds apply in the first place. Contextualised selection processes may be worthwhile and the evidence that individuals from non-selective schools outperform their independent school counterparts with the same A-level or equivalent grades [32] provides some rationale for varying requirements for entry based on socioeconomic background. From a widening participation perspective, more attention therefore needs to be paid to supporting the process of applicants “getting ready” (considering a medical career and preparing to apply) as well as “getting in” (what happens during selection from those who apply) [8, 16]. The large variation between medical schools in terms of the applicants they attract, and the proportion of applicants from low SES backgrounds who accept offers may indicate that some medical schools have implemented effective strategies to widen participation and better understanding of why these differences exist could help define and disseminate best practice. Second, there is no ideal measure of SES available. Consistent with our results, the experience of the Kings College London Extended Medical Degree Program (EMDP) illustrates this. EMDP applicants are drawn from schools with low average educational attainment in inner London, and represent a significantly broader demographic of applicants than non-EMDP, but one third of enrolled EMDP students were still from professional, middle class backgrounds [33]. Postcode and school type measures are also not straightforward to apply consistently across the four UK countries because the indices of multiple deprivation (IMD) used in each country are not strictly comparable [19] and school systems vary considerably. One reason this matters is because medical schools disproportionately recruit from their local populations, making publicly available league table comparisons of medical schools potentially problematic if based on inconsistent measures [34]. Individual measures like NS-SEC or an assessment of household income avoid this problem, but are not universally collected and the validity of data about parental occupation collected from teenagers is uncertain [26]. Additionally, were such data to be used in selection, then it would inevitably be open to gaming, although postcode of residence is not immune to this either. However, measuring the success or otherwise of widening participation will be difficult in the absence of consistent and robust measurement of individual SES, which will require gathering potentially sensitive data from all applicants and will have resource implications [3].

At present there is no set quota which medical schools must achieve in terms of recruiting for lower SES. However the Selection for Excellence Group (SEEG) set up by the Medical Schools Council have for the first time set ten year targets for medical schools aimed at increasing the percentage of students from a lower SES. These targets are based on the POLAR3 measure [35], which like IMD is a geographic measure, based on small area rates of participation by young people in higher education. It is therefore likely to have similar problems to IMD in relation to medical school applicants not necessarily sharing the characteristics of most residents of the postcode they live in. Indeed the SEEG report highlights that there are challenges with using this measure, including the fact that it is unable to provide a ‘granular view’ of geographic location [35]. Additionally like IMD, POLAR3 may not be directly comparable across the four UK countries due to differing definitions of what constitutes further or higher education. The SEEG report recommends that work is required to ‘develop additional targets for widening participation that utilise different data sets’. They also recommend that currently medical schools should be using ‘more than one source and different type of contextualised data in their admissions process’ and that they should ‘triangulate data to ensure the individuals they identify are truly from a widening participation background’. In the short term, medical schools are likely therefore to use the POLAR3 measure and to triangulate data such as parental occupation, IMD and school type in their admissions processes. Based on the results of this study and recommendations in the SEEG report, research is urgently required to develop a valid and reliable measure which can be used to compare applicants to and students accepted to medical schools across the four UK countries.

## Conclusion

Admission to medical school determines the composition of the medical profession in the future, and based on this analysis, medicine in the UK will remain dominated by those from more affluent backgrounds. There is no quick fix to widening participation, partly because gaining a place is rightly largely determined by academic ability. However, whether the future medical workforce will perform better or worse as a result of widening participation is unknown, requiring a consensus on which is the how best to measure SES within longitudinal research that tracks doctors across their careers.

## Abbreviations

IMD: Index of Multiple deprivation
NS-SEC: National Statistics Socio-Economic Classification
SES: socio-economic status
UKCAT: United Kingdom clinical aptitude test
